# Birth weight and prematurity with lung function at ~17.5 years: “Children of 1997” birth cohort

**DOI:** 10.1038/s41598-019-56086-7

**Published:** 2020-01-15

**Authors:** Baoting He, Man Ki Kwok, Shiu Lun Au Yeung, Shi Lin Lin, June Yue Yan Leung, Lai Ling Hui, Albert M. Li, Gabriel M. Leung, C. Mary Schooling

**Affiliations:** 1 School of Public Health, Li Ka Shing Faculty of Medicine, The University of Hong Kong, Hong Kong Special Administrative Region, China; 2 Department of Pediatrics, The Chinese University of Hong Kong, Hong Kong Special Administrative Region, China; 3 Graduate School of Public Health and Health Policy, City University of New York, New York, United States

**Keywords:** Risk factors, Epidemiology

## Abstract

We aimed to determine if prematurity and lower birth weight are associated with poorer lung function in a non-western developed setting with less marked confounding by socioeconomic position. Using multivariable linear regression in Hong Kong’s “Children of 1997” birth cohort, adjusted associations of prematurity and birth weight with forced expiratory volume in 1 second (FEV_1_), forced vital capacity (FVC), and forced expiratory flow at 25–75% of the pulmonary volume (FEF_25–75%_) at ~17.5 years were assessed. Associations for birth weight were stronger in boys for FEV_1_ (boys: 0.31 L, 95% confidence interval (CI) 0.24 to 0.38, girls: 0.18 L, 95% CI 0.12 to 0.25), FVC (boys: 0.36 L, 95% CI 0.27 to 0.44, girls: 0.22 L, 95% CI 0.15 to 0.28) and FEF_25–75%_ (boys: 0.35 L, 95% CI 0.21 to 0.49, girls: 0.22 L, 95% CI 0.09 to 0.34) adjusted for age, socioeconomic position and infant and maternal characteristics. Similarly adjusted, preterm birth (compared to full-term birth) was associated with lower FEV_1_/FVC and FEF_25–75%_. Thus, associations of lower birth weight, especially in boys, and prematurity with poorer lung function at 17.5 years were found. Identifying underlying mechanism might contribute to the improvement of pulmonary health and the prevention of adult respiratory illness.

## Introduction

The developmental origins of health and diseases (DOHaD) hypothesis emphasizes the role of poorer early growth, particularly in the first 1000 days, in non-communicable diseases (NCDs)^1^. Observationally poorer intrauterine experiences, proxied by birth weight or premature birth, are adversely associated with many aspects of adult health, including poorer lung function^2–6^. Lung function contributes both directly to chronic diseases via lung diseases and indirectly as a cardiovascular-related risk factor^7,8^, with potentially a causal role of forced expiratory volume^9^.

The DOHaD hypothesis has major implications for the care of mothers and babies. The DOHaD hypothesis largely rests on observational evidence from Western settings, which can be a very effective guide to action, for example as regards the harms of smoking^10^. On the other hand, there have been occasions when observational evidence has not been such a reliable guide, for example as regards the effects of hormone replacement therapy or vitamins^11,12^, likely because of residual confounding. Observational studies of birth attributes are open to confounding by maternal smoking, maternal overweight and lower socioeconomic position, which are also associated with worse birth outcomes and many health-conditions, including poorer lung function^13–16^. Birth weight also depends on gestational age which may not always be accurate, particularly in studies from before the routine use of ultrasound dating scans, when prematurity may be related to lower lung function^17–20^. The DOHaD hypothesis that early life is the critical period for respiratory health also does not consider evolutionary biology life history trade-offs, where early survival to reproductive age could trade-off against adult health including respiratory function. Lung function tracks throughout life^21^. Observationally, birth weight is inversely associated with indicators of restrictive lung function (forced vital capacity (FVC)) but evidence is weaker for the association of indicators of obstructive patterns (lower FEV_1_/FVC) in adults^5^. Previous studies have shown airway obstruction (lower FEV_1_, FEV_1_/FVC and forced expiratory flow between 25% and 75% of the pulmonary volume (FEF_25–75%_)) in extreme preterm births while fewer studies have focused on lung function in late preterm births^6,19,22–26^.

Evidence from twin studies, which are less open to confounding by family socio-economic position, is limited, but suggests lower birth weight is associated with poorer lung function, i.e. lower expiry in the first second of forced expiration (FEV_1_) and FVC^27–29^. More generally, Mendelian randomization studies of birth weight are difficult to interpret because of confounding by infant and/or maternal genetics^30,31^, but a study of monozygotic twins suggested little effect of birth weight on ischemic heart disease^32^. However, randomized controlled trials concerning the long-term effects of interventions targeting birth weight on lung function, are difficult and expensive to implement, and few such trials exist^33,34^. To our knowledge no Mendelian randomization studies have assessed whether birth weight is a causal factor for lung function, despite the relation between lung function and cardiovascular disease^7–9^. Hong Kong is a developed non-Western city, with social infrastructure and economic development similar to Western countries, where few women smoke, maternal overweight is less common than elsewhere^35^, and gestational age and birth weight are less confounded by socio-economic position^36^. Assessing the role of birth weight in lung function in this setting is less open to confounding possibly because of the less evident association of parental height with socioeconomic position in this population mainly formed by migrants from southern China in the mid-20^th^ century^37^. In this unique non-Western setting we examined the relations of birth weight and prematurity with lung function at ~17.5 years in a Hong Kong Chinese birth cohort: “Children of 1997”. We also considered whether the associations varied by sex, because of the shorter life expectancy in men than women.

## Methods

### Ethics statement

Ethical approval for the study, including comprehensive health related analyses, was obtained from the Institutional Review Board of the University of Hong Kong/Hospital Authority Hong Kong West Cluster (HKU/HA HKW IRB). Informed written consent was obtained from the parents/guardians, or from the participant if 18 years or older, before participation in the Biobank Clinical Follow-up. All methods were performed in accordance with the relevant guidelines and regulations.

### Data source

The current study utilizes a population-representative Chinese birth cohort from Hong Kong, “Children of 1997”, covering 88% of all births between April 1 and May 31, 1997, as described elsewhere^38^. At the infant’s first visit for free vaccinations and postnatal preventive care at all Maternal and Child Health Centers (MCHCs) in Hong Kong, 8237 mother-infant pairs were enrolled in an 18-month study to examine the association of environmental smoking with infant health^39^. Maternal characteristics (materal age at delivery and maternal smoking), family characteristics (education and residency status) and infant characteristics (birth weight, gestational age, parity and sex) were parent-or-career reported using a self-administered questionnaire in Chinese. Socioeconomic characteristics, such as parental occupation, household size and monthly household income were also reported. The study was resurrected as a birth cohort in 2005, with record linkage to Maternal and Child Health Center (MCHC) clinical records of growth and development including weight from birth to 5 years, and height from 3 months (with 96% success matching, n = 7999). In 2008–2012, active follow-up via three postal and/or telephone surveys was conducted. In 2013–2016, a Biobank clinical follow-up was conducted including anthropometrics, and a health check. Lung function was measured by spirometry (SpiroBank G Spirometer with WinspiroPRO software) and cleaned according to the American Thoracic Society/European Respiratory Society (ATS/ERS) criteria^40^. All lung function measurements were performed in a standing position with normal breath at rest before each test. Forceful slow inhalation and quick exhalation were performed not more than six times for at least three acceptable blows according to the ATS/ERS criteria, and the flow-volume curves with data were recorded. Any blow with a curve that did not resemble the ATS/ERS criteria predicted graph was considered as unacceptable and discarded. The spirometric curves with the maximum sum of FVC and FEV_1_ were selected.

### Exposures

Given the birth weight in our population representative cohort is lower than the WHO standard, the WHO standard is not suitable for measuring the body size at birth in our study, hence we considered birth weight as internal sex- and gestational age-specific z-scores (standard deviation score) for singletons in this population-representative birth cohort^41,42^. Gestational age was calculated from the interval between expected and actual date of delivery. Preterm birth was defined as birth before 37 completed gestational weeks. Using the most commonly used cutoffs (10th and 90th percentile in singletons), we categorized sex- and gestational age-specific z-score for birth weight into small-for-gestational age (SGA), appropriate-for-gestational age (AGA) and large-for-gestational age (LGA)^43^.

### Outcomes

The outcomes were lung function as FVC, FEV_1_, FEF_25–75%_ and FEV_1_/FVC, measured at ~17.5 years. Z-scores of these spirometric indices were based on the Global Lung Function Initiative (GLI) reference ^44^, which has specific age-, height- and sex- equations for South East Asians based on data from Hong Kong, southwest China, Taiwan and Thailand (collected in 1996–2002) and the Hong Kong reference ^45^.

### Statistical analysis

Baseline characteristics of the participants included and excluded were compared using Cohen’s *w* and Cohen’s *d* effect size, for categorical variables and continuous variables, where <0.1 and <0.2, respectively, indicate small differences between groups^46^. Analysis of variance (ANOVA) was used to compare birth weight, birth weight z-score and gestational age by potential confounders. Locally weighted scatterplot smoothing (LOWESS) curves were used to visualize the associations of birth weight and prematurity with lung function. Multivariable linear regression was used to examine adjusted associations of exposures and outcomes. Differences by sex were assessed from the significance of the relevant interaction term adjusted for confounding interactions. Potential confounders considered, i.e., common causes of birth weight or gestational age and lung function at ~17.5 years, included birth order, maternal birthplace, maternal smoking, maternal age at delivery, and parental socioeconomic position (including household income, highest parental occupation at recruitment and highest parental occupation). Potential mediators, such as breastfeeding, age of puberty, respiratory infections, weight and smoking, which might mediate the effect of birth weight on lung function but not cause birth weight were not included as confounders to avoid over adjustment^47^. To illustrate the potential effect of height, we present 2 models with different adjustment. Model 1 adjusted for confounders. Model 2 additionally adjusted for height in the sensitivity analysis using lung function in original units (Table S2). We only included singletons.

To predict missing values for gestational age (0.48% missing) and potential confounders (0.48% to 12.6% missing), multiple imputation based on additive regression and predictive mean matching was used. The regression model for multiple imputation incorporated exposures, outcomes and potential confounders, and interaction terms. To account for the probability of exclusion, inverse probability weighting was used to minimize possible selection bias induced by participants without valid lung function measurements. The inverse probability weights were calculated based on a logistic regression model with predictor variables including the exposures and the measured potential confounders after multiple imputation^48^. The single estimated *β* coefficients (mean difference) and 95% CI were summarized from 10 imputed datasets using Rubin’s rules and the inverse probability weights. Statistical analyses were conducted using R version 3.3.1 (R Foundation, Vienna, Austria).

## Results

As of January, 2017, 29 of the original 8327 cohort participants had permanently withdrawn and were excluded. Of the remaining 8298 participants, 6850 were considered potentially contactable for the Biobank clinical follow-up in 2013–2016. Of these, 3460 attended and completed the lung function test. Lung function curves for 415 participants failed the lung function acceptibility criteria and were excluded, leaving 3030 with valid lung function. Among these 3030, mean birth weight was 3156 grams for girls and 3234 grams for boys. Gestational age was 39.0 weeks on average, 136 (4.5%) were preterm with an average gestational age of 34.8 weeks, of whom 99 (72.8%) were born after 34 gestational weeks. Birth weight and gestational age in the participants with and without valid lung function data did not differ (Table 1). They also had similar maternal and socioeconomic characteristics (Table 1).

**Table 1:** Comparison of baseline characteristics for those with and without spirometry at ~17.5 years in Hong Kong’s “Children of 1997” birth cohort.

	Participants with spirometric data (N = 3033)		Participants without spirometric data (N = 5265)		Cohen’s *w*
	N	%	N	%	
Sex					0.03
Female	1475	49.0	2426	46.4	
Male	1522	51.0	2803	53.6	
Sex and gestational age adjusted birth weight Z-score					0.02
≤−2 SD	48	1.6	85	1.7	
−2 SD–1SD	390	13.1	699	13.7	
−1 SD −+1 SD	2100	70.4	3512	69.1	
+1 SD −+2 SD	361	12.1	629	12.4	
>2 SD	83	2.8	159	3.1	
Birth Order					<0.01
1	1403	47.5	2391	47.7	
2	1225	41.5	2065	41.2	
3 or above	323	10.9	556	11.1	
Preterm birth					0.03
Preterm	136	4.5	297	5.7	
Full-term	2883	95.5	4878	94.2	
Maternal age at delivery, years					0.07
≤24	294	9.7	714	14.0	
25–29	944	31.2	1568	30.9	
30–34	1176	38.9	1908	37.7	
≥35	607	20.1	873	17.2	
Mother’s birthplace					0.04
Hong Kong	1769	58.7	3018	62.6	
Mainland China	1247	41.3	1805	37.4	
Maternal smoking					0.04
No	2839	96.2	4722	94.2	
Yes	112	3.8	291	5.8	
Household income					0.04
1^st^ quintile	496	18.2	955	21.2	
2^nd^ quantile	552	20.2	932	20.7	
3^rd^ quantile	545	20.0	884	19.6	
4^th^ quantile	552	20.3	867	19.3	
5^th^ quantile	575	21.1	865	19.2	
Highest parental education					0.04
Grade 9 or below	861	28.5	1606	31.6	
Grade 10–11	1296	42.9	2151	42.4	
Grade 12 or above	863	28.6	1320	26.0	
Highest parental occupation					0.05
I (professional)	711	26.8	1003	22.7	
II (managerial)	392	14.8	730	16.5	
IIINM (non-manual skilled)	762	28.8	1284	29.1	
IIIM (manual skilled)	444	16.8	771	17.5	
VI (semiskilled)	256	9.7	470	10.7	
V (unskilled)	85	3.2	154	3.5	

Table 2 shows birth weight was positively associated with birth order, but was not clearly associated with maternal age or socioeconomic position. Lower gestational age was associated with higher parental education and older maternal age at delivery, but was not clearly associated with birth order, maternal smoking, household income or highest parental occupation. Those with native-born mothers had lower birth weight and gestational age than those whose mothers were born elsewhere.

**Table 2:** Baseline characteristics by gestational age, birth weight, and birth weight z-score adjusted for sex and gestational age in Hong Kong’s “Children of 1997” Birth Cohort.

Characteristics			Gestational age (week)			Birth weight (kg)			Birth weight z-score adjusted for sex and gestational age		
	No.	%	mean	SD	*P*	mean	SD	*P*	mean	SD	*P*
**Sex**											
Female	1488	49.0	39.1	1.53	<0.001	3.16	0.42	<0.001	−0.01	0.99	0.796
Male	1546	51.0	38.9	1.67		3.23	0.45		0.00	0.97	
**Birth order**											
1	1403	47.5	39.1	1.60	0.012	3.16	0.42	<0.001	−0.12	0.96	<0.001
2	1225	41.5	38.9	1.62		3.21	0.45		0.07	0.98	
3 or above	323	11.0	39.0	1.50		3.29	0.43		0.27	0.98	
**Maternal age at birth, years**											
≤24	294	9.7	39.3	1.69	<0.001	3.17	0.40	0.399	−0.16	0.88	0.004
25–29	944	31.2	39.1	1.60		3.21	0.42		−0.01	0.97	
30–34	1176	38.9	39.0	1.54		3.19	0.44		−0.01	0.97	
≥35	607	20.0	38.6	1.67		3.18	0.47		0.08	1.05	
**Maternal smoking**											
Smoker	112	3.8	39.1	1.81	0.385	3.16	0.41	0.422	−0.10	0.96	0.234
Non-smoker	2839	96.2	39.0	1.60		3.20	0.44		0.00	0.98	
**Maternal birthplace**											
Hong Kong	1769	58.7	38.8	1.59	<0.001	3.16	0.44	<0.001	−0.06	0.99	<0.001
Mainland China and elsewhere	1247	41.3	39.2	1.61		3.25	0.43		0.07	0.97	
**Household income (Quantile)**											
1	496	18.2	39.2	1.64	<0.001	3.23	0.42	0.093	0.03	0.97	0.469
2	552	20.3	38.9	1.71		3.20	0.47		0.03	1.03	
3	545	20.0	39.0	1.54		3.20	0.42		−0.01	0.96	
4	552	20.3	38.9	1.52		3.17	0.43		−0.06	1.01	
5	575	21.1	38.8	1.57		3.17	0.43		−0.04	0.94	
**Highest parental education at recruitment**											
Grade 9 or below	861	28.5	39.1	1.72	<0.001	3.23	0.44	0.062	0.05	0.98	0.082
Grade 10–11	1296	42.9	39.0	1.59		3.19	0.44		−0.04	1.00	
Grade 12 or above	863	28.6	38.8	1.50		3.18	0.43		−0.00	0.96	
**Highest parental occupation**											
I (professional)	711	26.8	38.9	1.55	<0.001	3.18	0.45	0.037	−0.05	0.94	0.012
II (managerial)	392	14.8	38.7	1.64		3.20	0.45		0.08	0.99	
IIINM (non-manual skilled)	762	28.8	39.0	1.58		3.17	0.43		−0.09	1.00	
IIIM (manual skilled)	444	16.8	39.2	1.62		3.24	0.43		0.06	1.04	
VI (semiskilled)	256	9.7	39.0	1.91		3.21	0.46		0.03	1.04	
V (unskilled)	85	3.2	39.2	1.60		3.28	0.44		0.18	1.04	

**Figure 1:**
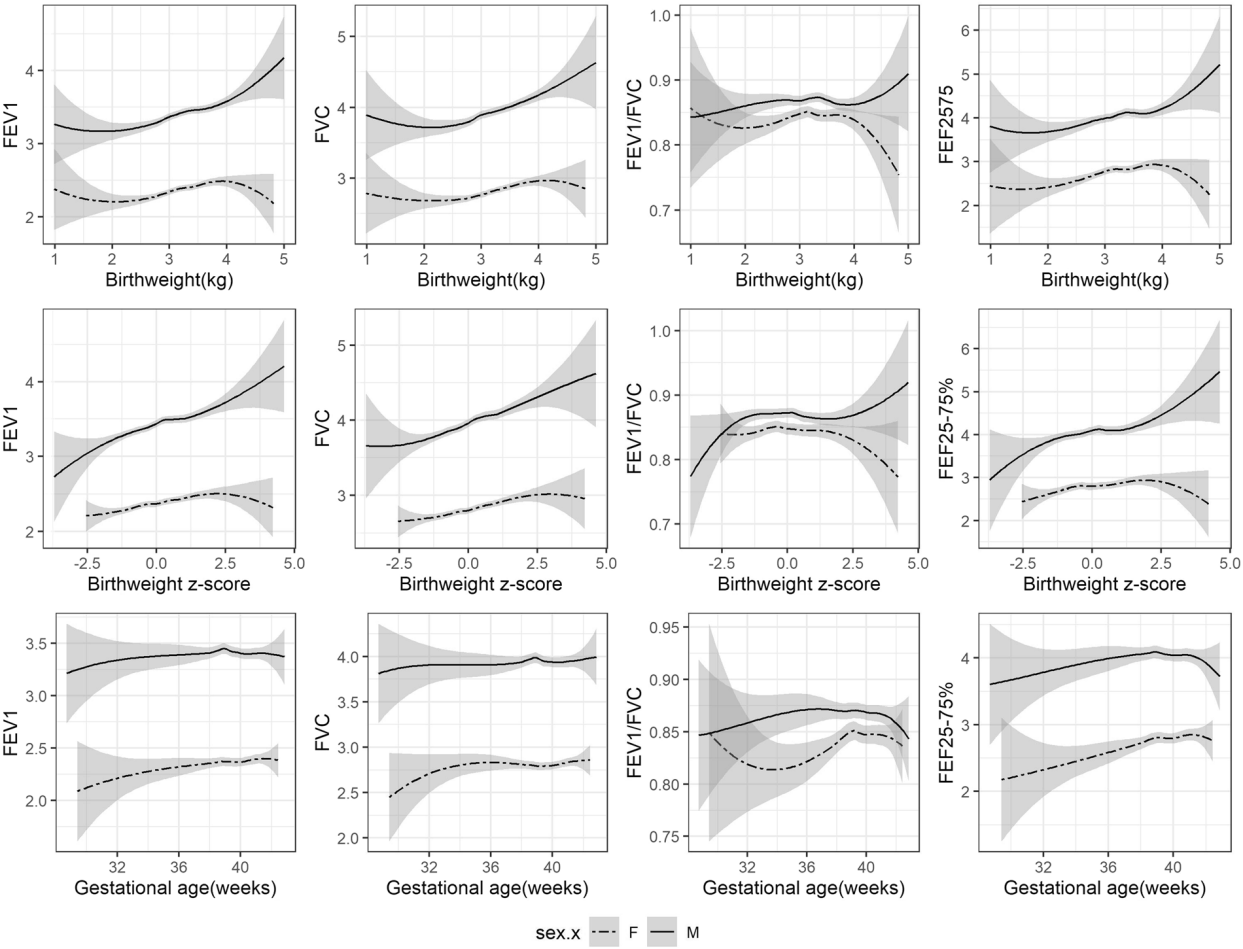
LOWESS curves showing association of birth weight, gestational age with lung function in original units by sex in Hong Kong’s “Children of 1997” Birth Cohort. Abbreviations: FEV_1_, forced expiratory volume in 1 second; FVC, forced vital capacity; FEF_25–75%_, forced expiratory flow at 25–75% of the pulmonary volume. Birth weight z-score refers to internal sex- and gestational age-specific z-scores.

**Figure 2:**
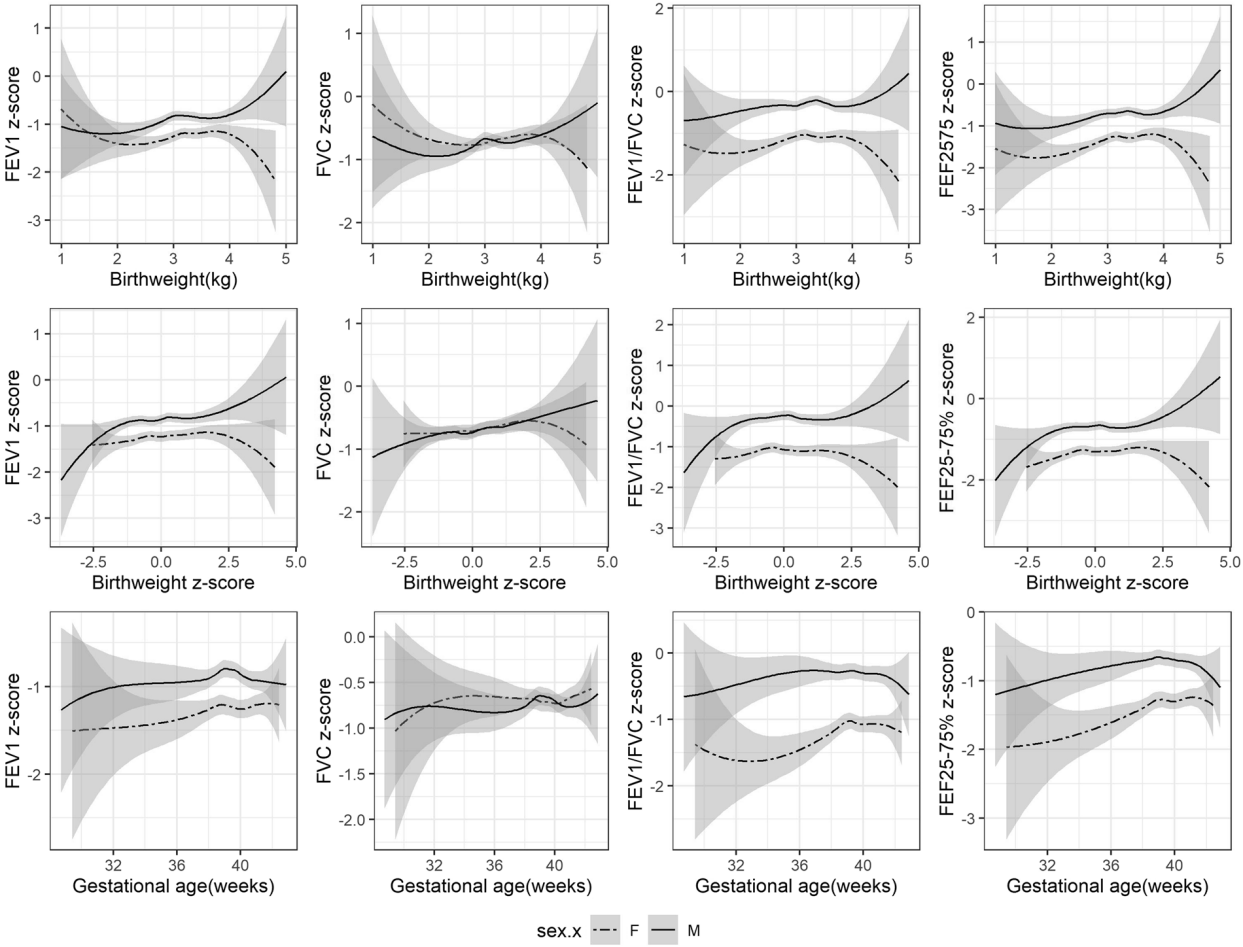
LOWESS curves showing association of birth weight, gestational ages with lung function in z-scores by sex in Hong Kong’s “Children of 1997” Birth Cohort. Abbreviations: FEV_1_, forced expiratory volume in 1 second; FVC, forced vital capacity; FEF_25–75%_, forced expiratory flow at 25–75% of the pulmonary volume. Birth weight z-scores refers to internal sex- and gestational age-specific z-scores. Lung function z-scores refers to age, height and sex specific z-scores based on the Global Lung Function Initiative (GLI) references.

Generally positive linear associations of birth weight in the normal range with FEV_1_, FVC and FEF_25–75%_ were found in boys and girls using LOWESS curves (Fig. 1), but not with FEV_1_/FVC ratio. Gestational age of less than 39 week was positively associated with FEV_1_/FVC ratio and FEF_25–75%_, but associations with FEV_1_ and FVC was less clear. The slope of birth weight for gestational age z-score with FEV_1_ and FVC was slightly steeper in boys than girls, with a significant sex-interaction (Table S1). Similar associations were seen for LOWESS curves of lung function using z-scores, but no clear difference by sex was seen for the slopes (Fig. 2). The associations of birth weight with FEV_1_ (p-value for interaction 0.02) and FVC (p-value for interaction 0.01) differed by sex, with stronger positive associations in boys than girls, adjusted for age, socioeconomic position and maternal and infant characteristics (Table 3). Birth weight was similarly positively associated with FEF_25–75%_ in boys and girls. Birth weight was not associated with FEV_1_/FVC ratio. Similarly, LGA (compared to AGA) was associated with higher FEV_1_, FVC and FEF_25–75%_ and SGA was associated with lower FEV_1_, FVC and FEF_25–75%_ but not with FEV_1_/FVC ratio. These associations were more evident in boys. This pattern remained after additionally adjusting for height, with the estimate slightly attenuated towards the null (Table S2). A consistent pattern of associations was found in the sex-specific analysis of lung function z-scores based on the sex-, age- and height specific equations from the GLI references (Table 4). The sex difference was not clear in analysis of z-scores (Table S1), although the positive associations of birth weight with FEV_1_ and FVC appeared stronger in boys than girls (Table 4).

**Table 3:** Associations of birth weight and gestational age with lung function in original units at ~17.5 years by sex in Hong Kong’s “Children of 1997” birth cohort (After inverse probability weighting and multiple imputation).

Sex	Exposures	FEV_1_, L				FVC, L				FEV_1_/FVC				FEF_25–75%_, L/s			
		Crude		Model 1^a^		Crude		Model 1^a^		Crude		Model 1^a^		Crude		Model 1^a^	
		β	95%CI	β	95%CI	β	95%CI	β	95%CI	β	95%CI	β	95%CI	β	95%CI	β	95%CI
**Girls**	Gestational age, w	0.02*	0.00 to 0.03	0.02*	0.00 to 0.03	0.01	−0.01 to 0.02	0.01	−0.01 to 0.02	0.00*	0.00 to 0.01	0.00*	0.00 to 0.01	0.05*	0.02 to 0.07	0.05*	0.02 to 0.07
	**Preterm**																
	Full term birth	Ref	—	Ref	—	Ref	—	Ref	—	Ref	—	Ref	—	Ref	—	Ref	—
	Preterm birth	−0.09	−0.20 to 0.02	−0.09	−0.21 to 0.02	0.00	−0.11 to 0.12	0.00	−0.12 to 0.11	−0.03*	−0.06 to −0.01	−0.03*	−0.06 to −0.01	−0.31*	−0.53 to −0.09	−0.31*	−0.53 to −0.09
	Birth weight, kg ^#^	0.17*	0.11 to 0.22	0.18*	0.12 to 0.25	0.18*	0.12 to 0.23	0.22*	0.15 to 0.28	0.01	−0.01 to 0.02	0.00	−0.01 to 0.01	0.26*	0.15 to 0.36	0.22*	0.09 to 0.34
	Birth weight z-score ^b #^	0.07*	0.05 to 0.09	0.07*	0.05 to 0.09	0.08*	0.06 to 0.11	0.08*	0.06 to 0.11	0.00	−0.01 to 0.02	0.00	−0.01 to 0.01	0.09*	0.04 to 0.13	0.08*	0.04 to 0.13
	**Size for gestational age**																
	AGA	Ref	—	Ref	—	Ref	—	Ref	—	Ref	—	Ref	—	Ref	—	Ref	—
	LGA	0.14*	0.06 to 0.21	0.14*	0.06 to 0.21	0.18*	0.11 to 0.26	0.18*	0.11 to 0.26	−0.01	−0.02 to 0.01	−0.01	−0.02 to 0.01	0.17*	0.02 to 0.31	0.16*	0.01 to 0.31
	SGA^#^	−0.11*	−0.19 to −0.03	−0.09*	−0.17 to −0.01	−0.11*	−0.19 to −0.03	−0.09*	−0.18 to −0.01	−0.01	−0.02 to 0.01	0.00	−0.01 to 0.01	−0.20*	−0.35 to −0.04	−0.18*	−0.34 to −0.02
**Boys**	Gestational age, w	0.00	−0.01 to 0.02	0.00	−0.01 to 0.02	0.00	−0.02 to 0.02	0.00	−0.02 to 0.02	0.00	0.00 to 0.00	0.00	0.00 to 0.00	0.02	−0.01 to 0.05	0.01	−0.02 to 0.04
	**Preterm**																
	Full term birth	Ref	—	Ref	—	Ref	—	Ref	—	Ref	—	Ref	—	Ref	—	Ref	—
	Preterm birth	−0.03	−0.14 to 0.08	−0.04	−0.15 to 0.08	0.00	−0.11 to 0.12	0.01	−0.12 to 0.14	−0.01*	−0.03 to 0.00	−0.01*	−0.03 to 0.00	−0.23*	−0.45 to −0.01	−0.23*	−0.45 to −0.01
	Birth weight, kg ^#^	0.22*	0.16 to 0.28	0.31*	0.24 to 0.38	0.25*	0.18 to 0.32	0.36*	0.27 to 0.44	0.00	−0.01 to 0.01	0.00	−0.01 to 0.01	0.28*	0.16 to 0.39	0.35*	0.21 to 0.49
	Birth weight z-score ^b #^	0.12*	0.09 to 0.15	0.12*	0.09 to 0.14	0.14	0.11 to 0.17	0.14*	0.11 to 0.17	0.00	0.00 to 0.00	0.00	0.00 to 0.00	0.13*	0.08 to 0.19	0.13*	0.07 to 0.18
	**Size for gestational age**																
	AGA	Ref	—	Ref	—	Ref	—	Ref	—	Ref	—	Ref	—	Ref	—	Ref	—
	LGA	0.21*	0.12 to 0.30	0.20*	0.11 to 0.30	0.22*	0.12 to 0.32	0.22*	0.11 to 0.32	0.00	−0.01 to 0.02	0.00	−0.01 to 0.02	0.30*	0.12 to 0.47	0.28*	0.11 to 0.45
	SGA^#^	−0.24*	−0.34 to −0.14	−0.24*	−0.34 to −0.14	−0.28*	−0.40 to −0.16	−0.28*	−0.39 to −0.16	0.00	−0.02 to 0.02	0.00	−0.02 to 0.02	−0.22*	−0.42 to −0.03	−0.21*	−0.40 to −0.01

Abbreviations: SGA, small-for-gestational age; AGA, appropriate-for-gestational age; LGA, large-for-gestational age; FEV_1_, forced expiratory volume in 1 second; FVC, forced vital capacity; FEF_25–75%_, forced expiratory flow at 25–75% of the pulmonary volume.

^a^Model 1: Adjusted for age, birth order, maternal age at birth, maternal smoking, maternal birthplace, and parental social-economic positions (SEP, including household income, the highest parental occupation at recruitment and the highest parental occupation).

^b^Apart from the adjustment in footnote a, adjusted for gestational age.

^c^For average gestational age (39 weeks), the mean for birthweight of girls and boys are 3377 grams and 3433 grams respectively, and the SD for birthweight of girls and boys are 374 grams and 421 grams respectively.

*Statistically significant association with lung function at the 0.05 level.

^#^Statistically significant interaction with sex on FEV_1_ and FVC.

**Table 4:** Associations of birth weight and gestational age with lung function in z-scores at ~17.5 years in Hong Kong’s “Children of 1997” birth cohort (After inverse probability weighting and multiple imputation).

Sex	Exposures	FEV_1_, z-score				FVC, z-score				FEV_1_/FVC, z-score				FEF_25–75%_, z-scores			
		Crude		Model 1^a^		Crude		Model 1^a^		Crude		Model 1^a^		Crude		Model 1^a^	
		β	95%CI	β	95%CI	β	95%CI	β	95%CI	β	95%CI	β	95%CI	β	95%CI	β	95%CI
**Both**	Gestational age, w	0.01	−0.02 to 0.03	0.01	−0.02 to 0.03	0.00	−0.02 to 0.03	0.00	−0.02 to 0.03	0.01	−0.02 to 0.04	0.02	−0.01 to 0.05	0.02*	0.00 to 0.05	0.02*	0.00 to 0.05
	**Preterm**																
	Full term birth	Ref	—	Ref	—	Ref	—	Ref	—	Ref	—	Ref	—	Ref	—	Ref	—
	Preterm birth	−0.07	−0.25 to 0.12	−0.07	−0.26 to 0.11	0.08	−0.10 to 0.26	0.07	−0.10 to 0.25	−0.26*	−0.48 to 0.04	−0.26*	−0.48 to −0.04	−0.26*	−0.46 to 0.05	−0.26*	−0.46 to −0.06
	Birth weight, kg ^b^	0.20*	0.11 to 0.29	0.26*	0.15 to 0.37	0.12*	0.03 to 0.21	0.18*	0.07 to 0.28	0.19*	0.08 to 0.30	0.22	−0.09 to 0.36	0.26*	0.16 to 0.37	0.31*	0.19 to 0.43
	Birth weight z-score ^c^	0.07*	0.03 to 0.12	0.07*	0.03 to 0.12	0.07*	0.03 to 0.11	0.07*	0.03 to 0.11	0.03	0.02 to 0.08	0.03	−0.02 to 0.08	0.07*	0.03 to 0.12	0.07*	0.02 to 0.12
	**Size for gestational age**																
	AGA	Ref	—	Ref	—	Ref	—	Ref	—	Ref	—	Ref	—	Ref	—	Ref	—
	LGA	0.12	−0.02 to 0.25	0.12	−0.02 to 0.26	0.13*	0.00 to 0.27	0.14*	0.00 to 0.27	0.04	−0.13 to 0.20	0.04	−0.13 to 0.20	0.15*	0.00 to 0.30	0.15*	0.00 to 0.30
	SGA^#^	−0.19*	−0.34 to −0.05	−0.19*	−0.34 to −0.04	−0.14	−0.29 to 0.01	−0.13	−0.28 to −0.01	−0.11	−0.29 to 0.07	0.10	−0.28 to 0.08	−0.20*	−0.36 to −0.03	−0.19*	−0.36 to −0.02
**Girls**	Gestational age, w	0.03	−0.01 to 0.07	0.03*	0.00 to 0.07	0.01	−0.03 to 0.04	0.01	−0.03 to 0.04	0.05*	0.01 to 0.10	0.06*	0.02 to 0.11	0.06*	0.02 to 0.10	0.07*	0.02 to 0.11
	**Preterm**																
	Full term birth	Ref	—	Ref	—	Ref	—	Ref	—	Ref	—	Ref	—	Ref	—	Ref	—
	Preterm birth	−0.21	−0.51 to 0.08	−0.23	−0.53 to 0.06	0.05	−0.22 to 0.33	0.03	−0.25 to 0.31	−0.48*	−0.82 to −0.14	−0.47*	−0.82 to −0.12	−0.31*	−0.53 to −0.09	−0.31*	−0.53 to −0.09
	Birth weight, kg ^b^	0.17*	0.03 to 0.31	0.17*	0.03 to 0.31	0.11	−0.02 to 0.25	0.11	−0.02 to 0.25	0.14	−0.02 to 0.31	0.14	−0.02 to 0.31	0.25*	0.10 to 0.41	0.25*	0.10 to 0.41
	Birth weight z-score ^c^	0.06*	0.00 to 0.12	0.06*	0.00 to 0.12	0.06*	0.00 to 0.12	0.06*	0.00 to 0.12	0.02	−0.05 to 0.09	0.02	−0.06 to 0.09	0.07*	0.01 to 0.14	0.07*	0.00 to 0.14
	**Size for gestational age**																
	AGA	Ref	—	Ref	—	Ref	—	Ref	—	Ref	—	Ref	—	Ref	—	Ref	—
	LGA	0.12	−0.08 to 0.31	0.11	0.09 to 0.31	0.19*	0.00 to 0.38	0.18	−0.01 to 0.37	−0.04	−0.27 to 0.19	−0.05	−0.28 to 0.19	0.12	−0.02 to 0.34	0.12	−0.10 to 0.34
	SGA^#^	−0.14	−0.35 to 0.06	−0.12	−0.33 to 0.09	−0.07	−0.27 to 0.13	−0.06	−0.25 to 0.14	−0.12	−0.36 to 0.13	−0.10	−0.35 to 0.14	−0.22	−0.44 to 0.01	−0.20	−0.43 to 0.03
**Boys**	Gestational age, w	0.00	−0.03 to 0.04	0.00	−0.03 to 0.03	0.00	−0.03 to 0.03	0.00	−0.04 to 0.03	0.01	−0.03 to 0.05	0.01	−0.03 to 0.05	0.02	−0.02 to 0.05	0.01	−0.02 to 0.05
	**Preterm**																
	Full term birth	Ref	—	Ref	—	Ref	—	Ref	—	Ref	—	Ref	—	Ref	—	Ref	—
	Preterm birth	−0.03	−0.25 to 0.20	−0.03	−0.26 to 0.20	0.10	−0.14 to 0.33	0.09	−0.15 to 0.32	−0.23	−0.50 to 0.04	−0.22	−0.50 to 0.05	−0.23*	−0.49 to 0.02	−0.24*	−0.49 to −0.02
	Birth weight, kg ^b^	0.16*	0.04 to 0.28	0.22*	0.07 to 0.36	0.13*	0.01 to 0.26	0.20*	0.05 to 0.35	0.08	−0.06 to 0.22	0.09	−0.08 to 0.27	0.17*	0.04 to 0.30	0.20*	0.03 to 0.36
	Birth weight z-score ^c^	0.08*	0.03 to 0.14	0.08*	0.02 to 0.13	0.08*	0.02 to 0.14	0.07*	0.02 to 0.13	0.03	−0.03 to 0.10	0.03	−0.04 to 0.10	0.07*	0.01 to 0.13	0.06*	0.00 to 0.12
	**Size for gestational age**																
	AGA	Ref	—	Ref	—	Ref	—	Ref	—	Ref	—	Ref	—	Ref	—	Ref	—
	LGA	0.12	−0.06 to 0.30	0.10	−0.08 to 0.29	0.08	−0.11 to 0.27	0.08	−0.11 to 0.27	0.11	−0.10 to 0.33	0.10	−0.12 to 0.32	0.18	−0.03 to 0.38	0.16	−0.04 to 0.36
	SGA	−0.21*	−0.41 to −0.01	−0.20*	−0.40 to 0.00	−0.21	−0.43 to −0.01	−0.20	−0.42 to 0.02	−0.03	−0.27 to 0.22	−0.02	−0.27 to 0.22	−0.12	−0.35 to 0.11	−0.11	−0.34 to −0.12

Abbreviations: SGA, small-for-gestational age; AGA, appropriate-for-gestational age; LGA, large-for-gestational age; FEV_1_, forced expiratory volume in 1 second; FVC, forced vital capacity; FEF_25–75%_, forced expiratory flow at 25–75% of the pulmonary volume.

^a^Adjusted for birth order, maternal age at birth, maternal smoking, maternal birthplace, and parental social-economic positions (SEP, including household income, the highest parental occupation at recruitment and the highest parental occupation).

^b^Apart from the adjustment in footnote a, adjusted for gestational age.

The associations of gestational age and preterm birth with lung function did not differ by sex, with no clear association of gestational age with lung function (Table S1). Preterm birth (compared to full-term birth) was associated with lower FEV_1_/FVC ratio and FEF_25–75%_ (Table 5). This pattern remained after additionally adjusting for height, with similar effect size (Table S2). Consistent results were found after converting lung function into z-scores (Table S4).

**Table 5:** Associations of birth weight and gestational age with lung function in original units at ~17.5 years in Hong Kong’s “Children of 1997” birth cohort (After inverse probability weighting and multiple imputation).

Exposures	FEV_1_, L				FVC, L				FEV_1_/FVC				FEF_25–75%_, L/s			
	Crude		Adjusted^a^		Crude		Adjusted^a^		Crude		Adjusted^a^		Crude		Adjusted^a^	
	β	95%CI	β	95%CI	β	95%CI	β	95%CI	β	95%CI	β	95%CI	β	95%CI	β	95%CI
Gestational age, w	−0.01	−0.03 to 0.00	0.01	0.00 to 0.02	−0.02	−0.03 to 0.00	0.01	−0.01 to 0.02	0.00	0.00 to 0.00	0.00	0.00 to 0.00	0.00	−0.02 to 0.03	0.03*	0.01 to 0.05
**Preterm**																
Full term birth	Ref	—	Ref	—	Ref	—	Ref	—	Ref	—	Ref	—	Ref	—	Ref	—
Preterm birth	0.03	−0.08 to 0.15	−0.06	−0.14 to 0.02	0.11	−0.02 to 0.24	0.01	−0.08 to 0.10	−0.02*	−0.03 to 0.00	−0.02*	−0.04 to −0.01	−0.15*	−0.34 to 0.03	−0.26*	−0.42 to −0.11
Birth weight, kg ^#^	0.30*	0.24 to 0.35	0.26*	0.21 to 0.30	0.33*	0.26 to 0.39	0.30	0.25 to 0.35	0.01	0.00 to 0.01	0.00	−0.01 to 0.01	0.39*	0.30 to 0.48	0.30*	0.20 to 0.39
Birth weight z-score ^b #^	0.10*	0.07 to 0.13	0.10*	0.08 to 0.11	0.12*	0.09 to 0.14	0.11	0.09 to 0.13	0.00	0.00 to 0.00	0.00	0.00 to 0.00	0.11*	0.07 to 0.15	0.11*	0.07 to 0.14
**Size for gestational age**																
AGA	Ref	—	Ref	—	Ref	—	Ref	—	Ref	—	Ref	—	Ref	—	Ref	—
LGA	0.17*	0.09 to 0.26	0.18*	0.12 to 0.24	0.20*	0.10 to 0.29	0.21*	0.14 to 0.27	0.00	−0.01 to 0.01	0.00	−0.01 to 0.01	0.23*	0.09 to 0.37	0.23*	0.12 to 0.35
SGA^#^	−0.22*	−0.31 to −0.13	−0.17*	−0.23 to 0.10	−0.24*	−0.35 to −0.14	−0.19*	−0.26 to −0.11	0.00	−0.02 to 0.01	0.00	−0.01 to 0.01	−0.27*	−0.41 to −0.12	−0.20*	−0.32 to −0.08

Abbreviations: SGA, small-for-gestational age; AGA, appropriate-for-gestational age; LGA, large-for-gestational age; FEV_1_, forced expiratory volume in 1 second; FVC, forced vital capacity; FEF_25–75%_, forced expiratory flow at 25–75% of the pulmonary volume.

^a^Adjusted for sex and confounders in Model 1: age, birth order, maternal age at birth, maternal smoking, maternal birthplace, and parental social-economic positions (SEP, including household income, the highest parental occupation at recruitment and the highest parental occupation).

^b^Apart from the adjustment in footnote a, adjusted for gestational age.

^c^For average gestational age (39 weeks), the mean for birthweight of girls and boys are 3377 grams and 3433 grams respectively, and the SD for birthweight of girls and boys are 374 grams and 421 grams respectively.

*Statistically significant association with lung function at the 0.05 level.

^#^Statistically significant interaction with sex on FEV_1_ and FVC.

## Discussion

In this developed non-Western setting with little confounding by socioeconomic position, inverse associations of birth weight with lung function, assessed by FEV_1_, FVC and FEF_25–75%_ at ~17.5 years were evident, particularly in boys. These findings are consistent with previous meta-analysis of observational studies from Western settings^4,5^ but add by showing boys to be more sensitive to birth weight. Given lung function z-scores are sex-specific, sex-differences in the associations of birth weight with lung function z-scores was attenuated. Similar to previous findings^4^, these associations remained after adjusting for height, suggesting that the association of birth weight with lung function is independent of height. Inconsistent association of birth weight with FEV_1_/FVC were observed in several previous studies^5^, while our study showed no association. Prematurity was inversely associated lower FEV_1_/FVC and FEF_25–75%_ and our previous study in this cohort showed preterm infants more prone to asthma^49^, these findings suggests impaired airway development in preterm births, which is consistent with other findings in late adolescence^19,20,22^.

In animal experiments, sheep and lambs with intrauterine growth restriction (IUGR) have structurally and functionally impaired lung development, suggesting that insults which cause IUGR may detrimentally affect the lungs resulting in persistent alternation in structure and function in later life^50–53^. Discrepancies also exist in the concentrations of surfactant proteins between IUGR and AGA human infants^54,55^. Alternatively, lower birth weight may represent less muscle mass even in adulthood which might reduce forced expiratory airflow^56,57^. Extremely premature newborns are born with fewer enlarged alveoli with thicker alveolar walls and hence their immature lung cannot function normally (with reduced effective gas exchange surface area) at birth, which may result in long-term lung functional abnormalities in later life^58^. Late preterm births are born at the late saccular stage of lung development when the lung volume and surface area is developing rapidly. Delivery during this period may also result in a less mature lung at birth and dysregulate alveolar development. Infants with immature lungs and poor airway function may be more susceptible to respiratory diseases after birth, which are also linked to worse lung function in late adolescence and early adulthood^17,18,59^. Other exposures that might lead to preterm birth, such as adverse maternal nutritional status, hypertensive disorders and overweight during pregnancy, might also affect airway development *in utero*^60^. Finally, poorer maternal lung function, for whatever reason, could result in both poorer birth outcomes and poorer offspring lung function. Apart from the negative exposure causing preterm birth, preterm births are more likely to receive neonatal intensive care, among which some interventions such as mechanical ventilation and oxygen therapy were associated with adverse respiratory health, abnormal lung growth and development^61^.

Stronger associations of birth weight with poor lung function in boys than girls have been observed before^62,63^ Boys are more vulnerable to respiratory diseases than girls^64,65^, but why this should affect particularly lower birth weight boys is unclear. Alternatively, androgens may adversely affect surfactant production coupled with the later start of pulmonary surfactant production in male fetuses^66^, which might make lighter boys more vulnerable. Finally lower birth weight may reduce muscle mass more in men than women^56,67^, possibly with corresponding effects on respiratory function via the respiratory muscles. Mechanistic studies are needed to distinguish between these possibilities so as to identify the best interventions to protect lung function in lower birth weight boys.

Although our study was conducted in a non-western setting with less obvious social patterning of birth weight and prematurity, several limitations exist. First, selection bias may exist, however, those with and without lung function had similar baseline characteristics (Table 1). Additionally, inverse probability weighting should help to recover the original sample based on measured covariates, thereby minimizing potential bias. Second, observational studies are open to unmeasured confounding, such as by air pollution^68^. Nevertheless, key confounders including maternal smoking and socioeconomic position were adjusted for in this study and hence our associations are unlikely to be explained by socioeconomic position or related attributes. Third, poorly reported gestational age without information from ultrasound scans might result in misclassification, which is likely to be small and non-differential because the information was reported by the main caregivers shortly after birth when gestational age looms large. Fourth, we did not assess potential mediators, such as diet and physical activity, and some potential parental confounders, such as maternal nutrition status. Fifth, the internal sex and gestational age specific z-scores for birth weight in the extremely preterm children (≤28 weeks) might be less accurate due to the small sample size of the very preterm births. Replication in other settings with birth weight and gestational age little confounded by socioeconomic factors, or re-examination using Mendelian Randomization accounting for maternal genetics is warranted to confirm the causal effects.

## Conclusions

In a population with minimal confounding by socioeconomic position, birth weight was inversely associated with FEV_1_, FVC and FEF_25–75%,_ particularly among boys, indicating lower birth weight may reduce lung function mainly in the airway capacity at 17.5 years. In contrast, prematurity was associated with lower FEV_1_/FVC and FEF_25–75%_, indicating preterm birth may impair airway development, which suggests increasing vulnerability to obstructive lung diseases. As such, our study suggests that both lower birth weight and prematurity may have long-lasting effects on lung function, which may be particularly detrimental for men.

## Supplementary information

Table S1-S2

## Data Availability

Data are available upon reasonable request from the “Children of 1997” data access committee: aprmay97@hku.hk.
