# Supplementary material for: Birth weight and prematurity with lung function at ~17.5 years: “Children of 1997” birth cohort
Source: Sci Rep. 2020 Jan 15;10:341. doi: 10.1038/s41598-019-56086-7 (PMC6962201; doi:10.1038/s41598-019-56086-7)
Supplement: Supplementary file 1 — Table S1-S2 [file 41598_2019_56086_MOESM1_ESM.docx]

**Birth weight and prematurity with lung function at ~17.5 years: “Children of 1997” birth cohort**

Baoting He, Man Ki Kwok, Shiu Lun Au Yeung, Shi Lin Lin, June Yue Yan Leung, Lai Ling Hui, Albert M Li, Gabriel M. Leung, and C. Mary Schooling^*^

**Supplementary tables**

Table S1. Exposures by sex interactions on lung function at ~17.5 years in the Hong Kong “Children of 1997” birth cohort. ^a^

1. Lung function z-scores

| **Interaction term** | **FEV_1, z-score_** | | **FVC, z-score** | | **FEV_1_/FVC, z-score** | | **FEF_25%-75%_** | |
| --- | --- | --- | --- | --- | --- | --- | --- | --- |
|  | **beta** | **P** | **beta** | **P** | **beta** | **P** | **beta** | **P** |
| Sex(boy) * birth weight ^b^ | 0.06 | 0.57 | 0.05 | 0.64 | 0.04 | 0.75 | 0.00 | 0.99 |
| Sex(boy) * gestational age | -0.02 | 0.26 | -0.02 | 0.59 | -0.04 | 0.11 | -0.06 | 0.42 |
| Sex(boy) * birth weight for gestational age z-score | 0.02 | 0.66 | 0.02 | 0.70 | 0.01 | 0.81 | -0.01 | 0.87 |
| Sex(boy) * preterm | 0.19 | 0.31 | 0.04 | 0.82 | 0.25 | 0.26 | 0.19 | 0.37 |
| Sex(boy) * LGA | 0.00 | 0.98 | -0.10 | 0.46 | 0.14 | 0.38 | 0.04 | 0.80 |
| Sex(boy) * SGA | -0.02 | 0.57 | -0.15 | 0.46 | 0.08 | 0.66 | 0.09 | 0.59 |

1. Lung function in original units

| **Interaction term** | **FEV_1_** | | **FVC** | | **FEV_1_/FVC** | | **FEF_25%-75%_** | |
| --- | --- | --- | --- | --- | --- | --- | --- | --- |
|  | **beta** | **P** | **beta** | **P** | **beta** | **P** | **beta** | **P** |
| Sex(boy) * birth weight ^b^ | 0.12 | 0.02* | 0.13 | 0.01* | 0.00 | 0.91 | 0.12 | 0.22 |
| Sex(boy) * gestational age | -0.01 | 0.25 | -0.01 | 0.63 | 0.00 | 0.11 | -0.03 | 0.13 |
| Sex(boy) * birth weight for gestational age z-score | 0.05 | 0.01* | 0.06 | <0.01* | 0.00 | 0.99 | 0.04 | 0.26 |
| Sex(boy) * preterm | 0.05 | 0.52 | 0.01 | 0.91 | 0.02 | 0.18 | 0.08 | 0.62 |
| Sex(boy) * LGA | 0.07 | 0.27 | 0.04 | 0.60 | 0.01 | 0.35 | 0.12 | 0.33 |
| Sex(boy) * SGA | -0.14 | 0.03* | -0.18 | 0.01* | 0.01 | 0.65 | -0.03 | 0.83 |

Abbreviations: SGA, small-for-gestational age; AGA, appropriate-for-gestational age; LGA, large-for-gestational age; FEV1, forced expiratory volume in 1 second; FVC, forced vital capacity; FEF25%-75%, forced expiratory flow at 25%-75% of the pulmonary volume.

a. Adjusted for age, birth order, maternal age at birth, maternal smoking, maternal birthplace, and parental social-economic positions (SEP, including household income, the highest parental occupation at recruitment and the highest parental occupation) and confounding interactions.

b. Apart from the adjustment in footnote a, adjusted for gestational age and corresponding confounding interactions.

Table S2. Direct associations of birthweight and gestational age with lung function at ~17.5 years stratified by sex in the Hong Kong “Children of 1997” birth cohort , independent of height and sex(After inverse probability weighting and multiple imputation),. ^a^

| **Sex** | **Exposures** | **FEV_1_, L** | | **FVC, L** | | **FEV_1_/FVC,%** | | **FEF_25%-75%_, L/s** | |
| --- | --- | --- | --- | --- | --- | --- | --- | --- | --- |
|  |  | **β** | **95%CI** | **β** | **95%CI** | **β** | **95%CI** | **β** | **95%CI** |
| **Both** | Gestational age, w | 0.01 | 0.00 to 0.02 | 0.00 | -0.01 to 0.01 | 0.00 | 0.00 to 0.00 | 0.03* | 0.01 to 0.05 |
|  | Preterm |  |  |  |  |  |  |  |  |
|  | Full term birth | Ref | - | Ref | - | Ref | - | Ref | - |
|  | Preterm birth | -0.04 | -0.11 to 0.04 | 0.03 | -0.05 to 0.11 | -0.02* | -0.04 to -0.01 | -0.25* | -0.40 to -0.10 |
|  | Birth weight, kg ^b^ | 0.13* | 0.08 to 0.18 | 0.14* | 0.09 to 0.19 | 0.00 | -0.01 to 0.01 | 0.20* | 0.11 to 0.30 |
|  | Birth weight z-score ^c^ | 0.05* | 0.03 to 0.07 | 0.05* | 0.03 to 0.07 | 0.00 | 0.00 to 0.00 | 0.07* | 0.04 to 0.11 |
|  | Size for gestational age |  |  |  |  |  |  |  |  |
|  | AGA | Ref | - | Ref | - | Ref | - | Ref | - |
|  | LGA | 0.09* | 0.03 to 0.14 | 0.10* | 0.03 to 0.16 | 0.00 | -0.01 to 0.01 | 0.17* | 0.05 to 0.28 |
|  | SGA | -0.04 | -0.11 to 0.04 | -0.09* | -0.16 to-0.03 | 0.00 | -0.02 to 0.01 | -0.14* | -0.27 to -0.02 |
| **Girls** | Gestational age, w | 0.01 | -0.00 to 0.03 | 0.00 | -0.01 to 0.02 | 0.00 | 0.00 to 0.01 | 0.05* | 0.02 to 0.07 |
|  | Preterm |  |  |  |  |  |  |  |  |
|  | Full term birth | Ref | - | Ref | - | Ref | - | Ref | - |
|  | Preterm birth | -0.08 | -0.19 to 0.03 | 0.02 | -0.10 to 0.12 | -0.03* | -0.06 to -0.01 | -0.23* | -0.45 to -0.01 |
|  | Birth weight, kg ^b^ | 0.08* | 0.03 to 0.14 | 0.09* | 0.03 to 0.14 | 0.01 | -0.01 to 0.02 | 0.18* | 0.07 to 0.29 |
|  | Birth weight z-score ^c^ | 0.03* | 0.01 to 0.06 | 0.04* | 0.02 to 0.07 | 0.00 | -0.01 to 0.00 | 0.05* | 0.00 to 0.10 |
|  | Size for gestational age |  |  |  |  |  |  |  |  |
|  | AGA | Ref | - | Ref | - | Ref | - | Ref | - |
|  | LGA | 0.07 | -0.01 to 0.14 | 0.11* | 0.04 to 0.19 | -0.01 | -0.03 to 0.01 | 0.10 | -0.05 to 0.25 |
|  | SGA | -0.05 | -0.13 to 0.03 | -0.04* | -0.12 to -0.04 | -0.00 | -0.02 to 0.01 | -0.13 | -0.29 to 0.03 |
| **Boys** | Gestational age, w | 0.00 | -0.02 to 0.02 | 0.00 | -0.02 to 0.02 | 0.00 | -0.02 to 0.00 | 0.01 | -0.02 to 0.04 |
|  | Preterm |  |  |  |  |  |  |  |  |
|  | Full term birth | Ref | - | Ref | - | Ref | - | Ref | - |
|  | Preterm birth | -0.02 | -0.13 to 0.09 | 0.04 | -0.08 to 0.16 | -0.01 | -0.03 to 0.00 | -0.23* | -0.44 to -0.01 |
|  | Birth weight, kg ^b^ | 0.11* | 0.05 to 0.17 | 0.11* | 0.04 to 0.17 | 0.01 | -0.01 to 0.01 | 0.21* | 0.09 to 0.32 |
|  | Birth weight z-score ^c^ | 0.06* | 0.04 to 0.09 | 0.06* | 0.03 to 0.09 | 0.00 | -0.00 to 0.01 | 0.10* | 0.04 to 0.15 |
|  | Size for gestational age |  |  |  |  |  |  |  |  |
|  | AGA | Ref | - | Ref | - | Ref | - | Ref | - |
|  | LGA | 0.12* | 0.03 to 0.20 | 0.09 | -0.01 to 0.19 | 0.01 | -0.01 to 0.02 | 0.23* | 0.06 to 0.41 |
|  | SGA | -0.13* | -0.23 to -0.03 | -0.12* | -0.23 to -0.01 | -0.00 | -0.02 to 0.01 | -0.14 | -0.34 to 0.06 |

Abbreviations: SGA, small-for-gestational age; AGA, appropriate-for-gestational age; LGA, large-for-gestational age; FEV_1_, forced expiratory volume in 1 second; FVC, forced vital capacity; FEF_25%-75%_, forced expiratory flow at 25%-75% of the pulmonary volume.

a Adjusted for height and confounders in Model 1:age, birth order, maternal age at birth, maternal smoking, maternal birthplace, and parental social-economic positions (SEP, including household income, the highest parental occupation at recruitment and the highest parental occupation).

b Apart from the adjustment in footnote a, adjusted for gestational age.

c For average gestational age (39 weeks), the mean for birthweight of girls and boys are 3377 grams and 3433 grams respectively, and the SD for birthweight of girls and boys are 374 grams and 421 grams, respectively.
